# Complete genome sequence of *Pediococcus pentosaceus* TOKAI 759m, a promising soy milk yogurt starter isolated from rice in Japan

**DOI:** 10.1128/mra.00703-25

**Published:** 2025-10-29

**Authors:** Tomoyuki Hibi, Takahiro Kawanabe, Yuki Nakashima, Shin Yasuda, Masashi Hirano, Hideki Kinoshita

**Affiliations:** 1 Graduate School of Bioscience, Tokai University 208559 , Mashiki-machi, Kumamoto, Japan; 2 Research Institute of Agriculture, Tokai University 208559 , Mashiki-machi, Kumamoto, Japan; 3 Undergraduate School of Agriculture, Tokai University 212250, Mashiki-machi, Kumamoto, Japan; 4 JSPS Research Fellowship for Young Scientists, Tokai University 212250, Mashiki-machi, Kumamoto, Japan; 5 Probio Co. Ltd., Nishihara-mura, Kumamoto, Japan; University of Pittsburgh School of Medicine, Pittsburgh, Pennsylvania, USA

**Keywords:** complete genome, lactic acid bacteria, *Pediococcus pentosaceus*, soy milk yogurt starter, Illumina, Oxford Nanopore Technologies

## Abstract

*Pediococcus pentosaceus* TOKAI 759m is a Gram-positive bacterium isolated from rice in Japan. Our previous study demonstrated that this strain is a promising starter culture for soy milk yogurt due to its various beneficial properties. The strain has been commercialized. In this study, we present the complete genome sequence of this strain.

## ANNOUNCEMENT

Our previous study demonstrated that the soy milk yogurt fermented with *Pediococcus pentosaceus* TOKAI 759m (TK 759m) can modulate the gut microbiota and potentially suppress the production of pro-inflammatory cytokines (1) and neuroinflammation (2) *in vivo*. TK 759m was originally isolated from rice in Japan (3) and has recently been commercialized with a product name “Soypedio”. Briefly, washed rice water was plated on De Man, Rogosa, and Sharpe (MRS) (Difco Laboratories, Detroit, MI, USA) agar at 37°C for 2 days under anaerobic conditions. A single colony was then inoculated in MRS broth and cultured at 37°C for 24 h. After several passages, the strain was stored at −80°C in glycerol.

**Fig 1:**
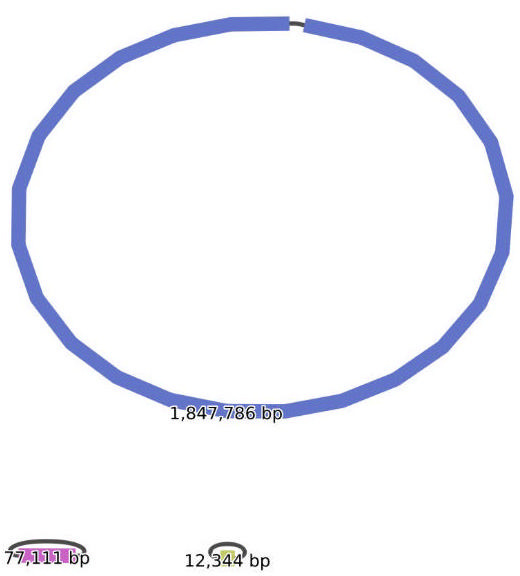
Bandage plot of complete genome assembly.

TK 759m was cultured overnight under the same conditions described above. Following incubation, the bacterial cells were harvested by centrifugation. First, pellets intended for short-read sequencing were sent to MySkin Corporation (Tokyo, Japan). Genomic DNA was then extracted from the pellet using DNeasy Blood & Tissue Kit (QIAGEN, Hilden, Germany) according to the manufacturer’s protocol. Sequencing libraries were prepared using MiSeq Reagent Kit v3 (Illumina, San Diego, CA, USA), and 300 bp paired-end reads were generated on a MiSeq platform (Illumina). The raw short reads were quality-checked and trimmed using fastp v.0.24.0 (4, 5) with the parameters “-q 30 -n 20 -t 1 -T 1.” Before trimming, a total of 3,657,140 reads were obtained, with a combined total sequencing of 611,318,165 bp. Meanwhile, genomic DNA for long-read sequencing was extracted from the pellet using Wizard Genomic DNA Purification Kit (Promega, Madison, WI, USA). No size selection of DNA was performed before library preparation. Libraries were prepared using Rapid Sequencing Kit V14 (SQK-RAD114, Oxford Nanopore Technologies [ONT], Oxford, UK) and sequenced on R10.4.1 Flow Cell (FLO-FLG114, ONT) on a MinION Mk1B (ONT) under the control of MinKNOW v.24.11.10 with default settings. The sequence data were then basecalled with Dorado v.7.6.8 (High-accuracy model v.4.3.0, 400 bps), which is built into the MinKNOW software. The raw long reads were assessed using NanoPlot v1.44.1 (6). The read N50 was 17,625 bp, and the total number of raw reads was 18,109 reads. Subsequently, the raw long reads were trimmed using NanoFilt v.2.8.0 (7) with the parameters “-q 10 --headcrop 50 -l 1000”. Trimmed long and short reads were assembled using Unicycler v0.5.1 (8) with default settings. Circularization of each contig was determined using Bandage v0.8.1 (Fig. 1; 9). Trimmed short reads were then mapped to assembled sequences with BWA-MEM2 v2.2.1 (10), indexed using SAMtools v1.21 (11). Finally, the assembled sequences were polished using indexed short reads with Pilon v1.24 (12). This polishing workflow was repeated once.

Completeness check, taxonomy check, and annotation of polished sequences were performed using DDBJ Fast Annotation and Submission Tool (DFAST) v1.3.6 (13). In this annotation, the genome was rotated to start with the *dnaA* gene. Final genome coverage was calculated by mapping the trimmed short and long reads to the annotated FASTA file using minimap2 v2.29 (14, 15) and SAMtools v1.21 (11). Detailed information on the annotated genome is shown in Table 1.

**TABLE 1:** Accession numbers and sequencing statistics for *P. pentosaceus* TOKAI 759m

Assembly statistics	Value
Total length (bp)	1,937,242
No. of sequences	3
Chromosome mean depth (×)	338.572
Plasmid 1 mean depth (×)	327.538
Plasmid 2 mean depth (×)	767.818
GC content (%)	37.4
No. of CDSs	1916
No. of rRNAs	15
No. of tRNAs	56
No. of CRISPRS	1
Completeness check (%)	93.67
Taxonomy check (%) (ANI to *Pediococcus pentosaceus* NBRC 107768 [GCA_007992275.1])	98.92

## Data Availability

All data sets have been deposited in DDBJ/ENA/GenBank under the following accession numbers: BioProject PRJDB35442, BioSample SAMD01046883, raw short-read data DRR698283, raw long-read data DRR698284, and assembled and annotated read data AP041211, AP041212, and AP041213.
